# The metastasis suppressor CD82/KAI1 regulates cell migration and invasion via inhibiting TGF-β 1/Smad signaling in renal cell carcinoma

**DOI:** 10.18632/oncotarget.18086

**Published:** 2017-05-23

**Authors:** Jundong Zhu, Chao Liang, Yibo Hua, Chenkui Miao, Jianzhong Zhang, Aiming Xu, Kai Zhao, Shouyong Liu, Ye Tian, Huiyu Dong, Chao Zhang, Pu Li, Shifeng Su, Chao Qin, Zengjun Wang

**Affiliations:** ^1^ Department of Urology, The First Affiliated Hospital of Nanjing Medical University, Nanjing, China

**Keywords:** CD82/KAI1, tetraspanin, migration/invasion, TGF-β1/Smad signaling, renal cell carcinoma

## Abstract

The tetraspanin KAI1/CD82 was identified as a tumor metastasis suppressor that downregulated in various malignant cell types. However, the function of CD82 and its underlying anti-metastasis role in renal cell carcinoma (RCC) is still unraveled. Here, we investigated the expression of CD82 in RCC and explored its regulatory mechanism in RCC cell lines. We found that CD82 was down-regulated in RCC tissues and cells and its expression was significantly associated with histological grade(p=0.041), tumour stage (p=0.036) and tumor size(p=0.020) by analyzing tissue microarrays. After upregulation of CD82 through lentivirus, reduced ability of migration and invasion in Caki-1 cells were detected. In contrast, gene silencing of CD82 by small interfering RNA promoted metastatic and invasive potential of 786-O cells. Furthermore, Western blot was performed to identify the influence of CD82 on MMP family and TGF-β1/Smad pathway in RCC. Subsequently, upregulating protein level of TGF-β1 with the overexpression of CD82 could rescue the malignant behaviors inhibited by CD82 which indicated that CD82 played its inhibitory role in RCC partially by attenuating the expression of TGF-β1. Taken together, CD82 played a prominent role in migration and invasion of RCC cells and it might exhibit its inhibitory role in RCC metastasis via block TGF-β1/Smad signaling pathway.

## INTRODUCTION

Kidney cancer is one of the top 10 most common cancers in the world and renal cell carcinoma (RCC) accounts for 85% of all renal malignancies with an incidence that continues to rise. It is estimated that 320,000 new cases will be diagnosed in 2016 and the globally estimated number of deaths is 140,000 [1]. The most effective way to cure RCC is tumor resection because of its resistance to conventional chemotherapy and radiotherapy [2–4]. Although surgery can cure early stage RCC, nearly 20-40% of patients still develop metastasis or locally recurrent after nephrectomy, with only a median survival of 6-12 months and a 5-year survival of 9% [5]. Therefore, exploring potential molecular mechanisms and identifying new therapeutic targets are becoming extremely necessary for RCC clinical treatment.

CD82, also known as KAI-1, belongs to tetraspanin family associated not only with extensive physiological processes, but also in pathological situations such as cancer invasion and metastasis [6, 7]. Dong et al. firstly identified the location of CD82 gene on chromosome 11p and showed it could suppress metastasis when introduced into rat AT6.1 prostate cancer cells [8]. Recent years studies have demonstrated CD82 as a metastatic suppressor in many malignant solid cancer cell types including prostate cancer [9],bladder cancer [10], breast cancer [11], pancreatic cancer, and hepatocarcinoma [12] cells. Current understanding of CD82 function indicates it is likely to be involved in detachment, motility/invasion, and cell survival, which are associated with various adhesion receptors (eg integrins), receptor tyrosine kinases (eg EGFR and c-Met) and other signalling pathway molecules [6, 13, 14]. Despite wide investigations, few researches reported how CD82 specifcally affects RCC metastasis and the underlying molecular mechanism of CD82 in RCC is not yet fully determined.

In this study, to investigated the potential of CD82 on RCC metastasis in RCC cells, lentiviral vector and small interfering RNAs (siRNAs) were used to overexpress or silence CD82 in RCC cell lines. What's more, we found the TGF-β1 pathway was involved in the metastasis of RCC cell lines. The following results may revealed how CD82 act as a tumor suppressor in RCC cell lines and provide a potential biomarker for treatment of RCC.

## RESULTS

### Downregulation of CD82 in RCC tissues and cells

**Figure 1:**
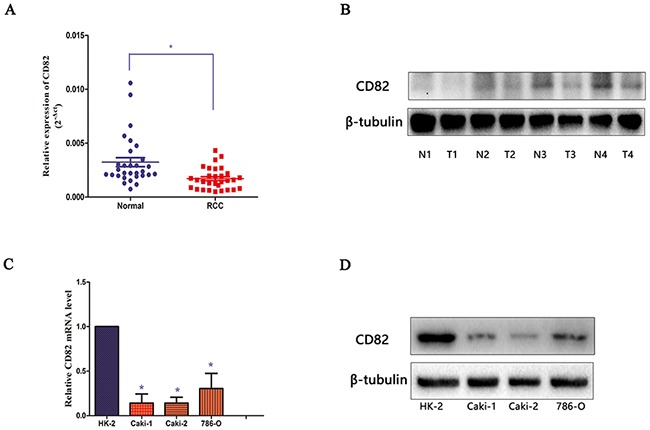
CD82 is downregulated in RCC tissues and cells CD82 level in RCC samples was significantly downregulated compared with the paired adjacent non-tumor tissues according to qRT-PCR data **(A)** and immunoblotting **(B)** respectively. CD82 expression in RCC cell lines (Caki-1, Caki-2, 786-O) and renal tubular epithelial cell (HK-2) was also tested by qRT-PCR **(C)** and immunoblotting **(D)** methods. The median in each triplicate was used to calculate the relative CD82 mRNA expression using the comparative 2^-ΔΔCt^ method. All data are presented as mean± SD. * P < 0.05 compared with the adjacent non-tumor tissues or HK-2 cell line.

The mRNA expression of CD82 was examined in 30 paired clinical RCC tissues and their adjacent normal tissues by qPCR. The results showed that CD82 was significantly down-regulated in RCC tissues, compared with adjacent normal tissues (p<0.05, Figure 1A). Then, we extracted the protein from the tissues and western blot analyses confirmed down-regulated levels of CD82 in RCC tissues, compared with normal tissues (Figure 1B). In addition, we tested the mRNA levels of CD82 in three RCC cell lines including Caki-1, Caki-2 and 786-O. The results showed that CD82 mRNA and protein expression were both significantly higher in HK-2 cell line (Figure 1C, 1D). All the results confirmed the down-regulated levels of CD82 in RCC tissues and cell lines.

### Inhibition of CD82 on cell migration and invasion

**Figure 2:**
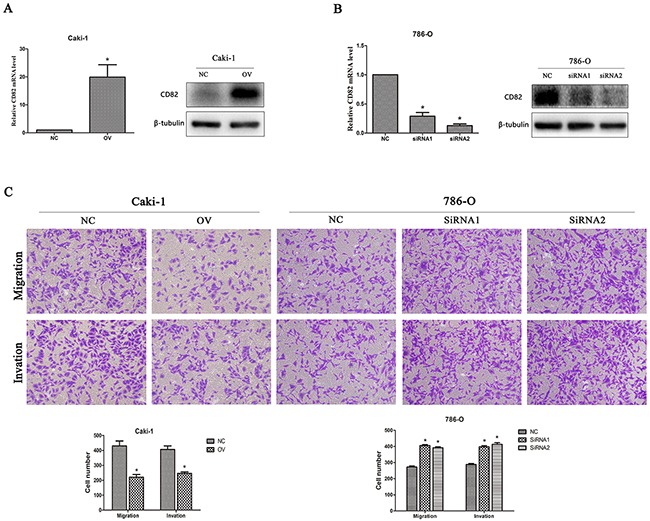
CD82 inhibits cell migration and invasion in RCC cells Relative expression of CD82 in transfected cells. The expression of CD82 was upregulated in transfected Caki-1 cells **(A)** and downregulated by two SiRNA in 786-O cells **(B)**. * P < 0.05 compared with the negative control group. **(C)** Overexpression of CD82 inhibited migration and invasion in Caki-1 cell line and knockdown of CD82 significantly promoted the migration and invasion of 786-O cells. Data are mean ± SD of at least three independent experiments. * P < 0.05 compared with the negative control group. Original magnification 200×.

To study the functional role of CD82 in RCC cells, CD82 was blocked down in 786-O by two siRNA and overexpressed in CAKI-1 by transfecting lentiviral vector. As shown in Figure 2, CD82 mRNA and protein expression were highly upregulated in Caki-1(Figure 2A) and significantly downregulated in 786-O (Figure 2B) after transfection. Migration and invasion assays were performed to explore whether CD82 affects migration and invasion abilities in Caki-1 and 786-O. Compared with negative control, overexpression of CD82 inhibited the migration and invasion of Caki-1 cells. Conversely, knockdown of CD82 significantly promoted the migration and invasion of 786-O cells (Figure 2C). These results suggested that CD82 may inhibit RCC cells metastasis.

### CD82 on the regulation of TGF-β1 signaling

**Figure 3:**
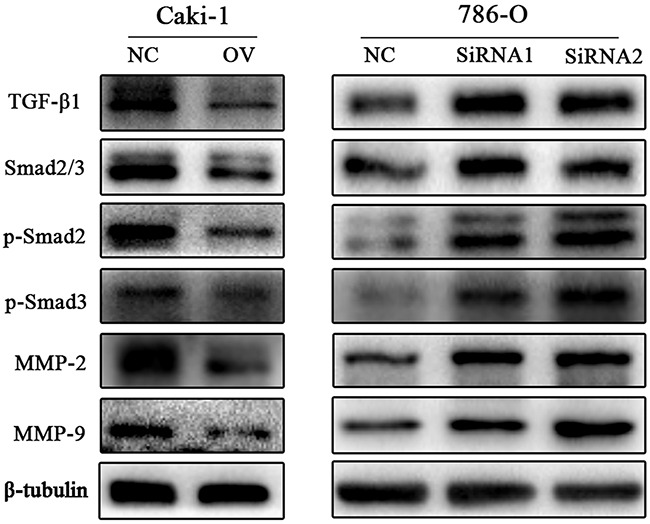
CD82 suppresses the TGF-β1/Smad/MMP pathway Western blot analysis was used to detect the changes in MMP family and TGF-β1/Smad pathway protein expression after changing CD82 expression. In Caki-1 cells, MMP-2, MMP-9, TGF-β1 were decreased with a high expression of CD82 and the loss of p-Smad2 and p-Smad3, downstream of TGF-β1, were observed in OV group cells. The opposite results was found in SiRNA transfected 786-O cells. *P < 0.05 compared with the negative control group.

To explore the mechanism of CD82 on RCC cells migration and invasion, we assessed the effect of CD82 expression on the migration pathways-related protein including matrix metalloproteinase MMP-2 and MMP-9 in Caki-1 and 786-O cells. The expression of MMP2 and MMP9 were significant decreased with overexpression of CD82 in Caki-1 cell line and increased in 786-O cell line 48 h after transfection of siCD82. We also tested the protein expression of TGF-β1, a regulator of MMPs, and the data showed that overexpression of CD82 notably restrained the protein expression levels of TGF-β1 and the opposite results were found in CD82-knockdown cell line. Then, we detected the phosphorylation of Smad2 and Smad3(p-Smad2, p-Smad3), downstream of TGF-β1 by immunoblotting. Transfection of CD82 lentiviral in Caki-1 cells and siRNA in 786-O cells remarkably affected the phosphorylation of Smad2 and Smad3 in consistent with the changes of TGF-β1. (Figure 3)

### Rescue experiment on cell migration and invasion

**Figure 4:**
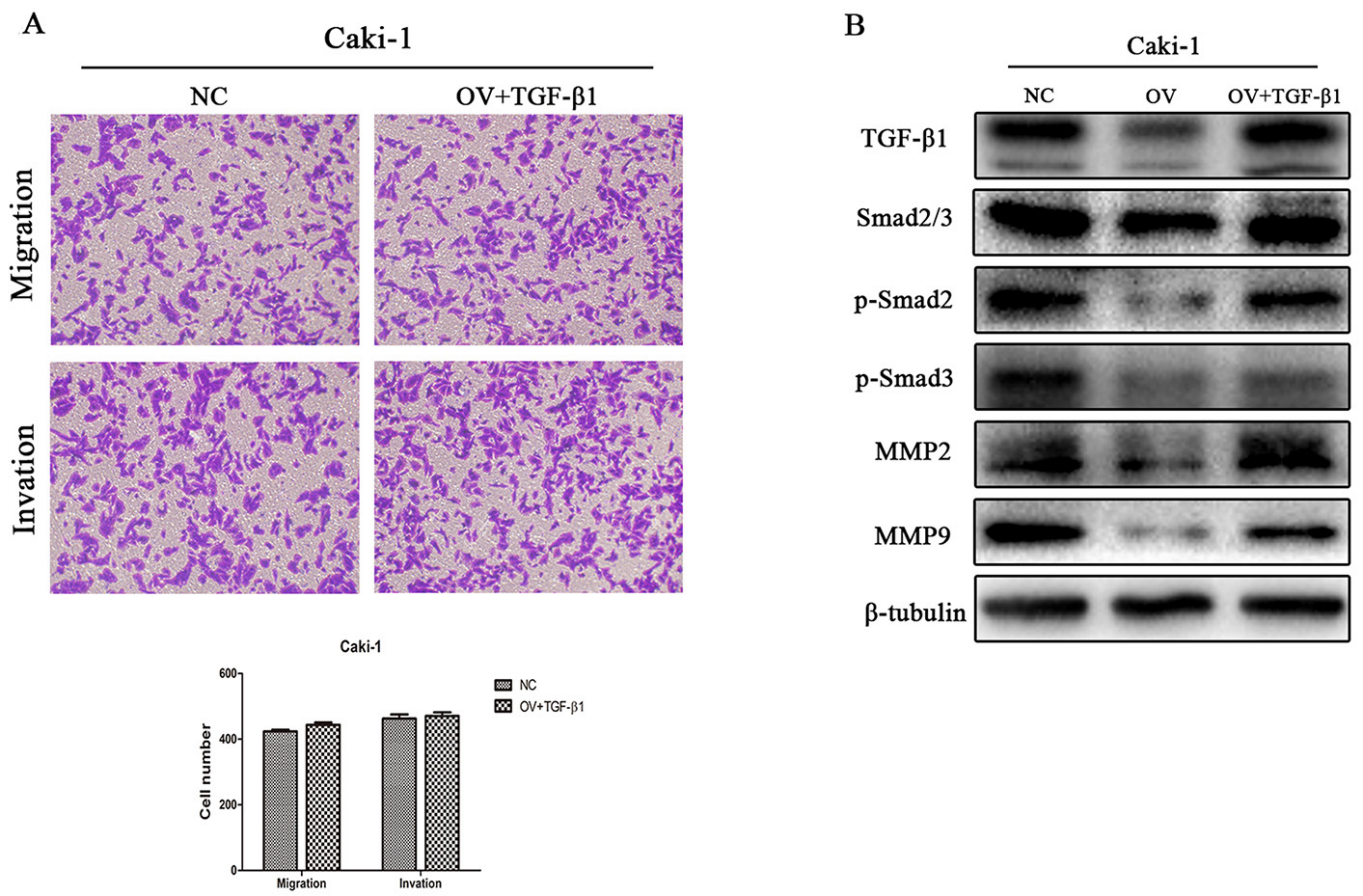
Observation on cell migration and invasion after Rh TGF-β1 stimulation in OV group cells Inhibitory role of CD82 on migration and invasion of Caki-1 cells was attenuated after Rh TGF-β1 stimulation **(A)**. TGF-β1 protein expression in Caki-1 cells, as well as p-Smad2, p-Smad3, MMP2 and MMP9 was restored after the stimulation **(B)**. Data are mean ± SD of at least three independent experiments. P >0.05 compared with the negative control group.

Using the transwell assays described previously, decreased migration and invasion capabilities induced by CD82 overexpression were alleviated after Rh TGF-β1 stimulating. The results showed no significant difference between NC group and OV+TGF-β 1 group (p>0.05, Figure 4A). After Rh TGF-β1 stimulating, proteins were isolated from cells and we detected the expression of TGF-β1, MMP2,MMP9, as well as the phosphorylation of Smad2 and Smad3 by immunoblotting. As shown in Figure 4B, TGF-β1 protein expression was upregulated in Caki-1 cells and the levels of p-Smad2, p-Smad3, MMP2 and MMP9 were restored subsequently. These results indicated that CD82 may inhibit cell migration and invasion in RCC partially by attenuating the expression of TGF-β1.

### Relationship between CD82 and clinic factors of RCC

**Figure 5:**
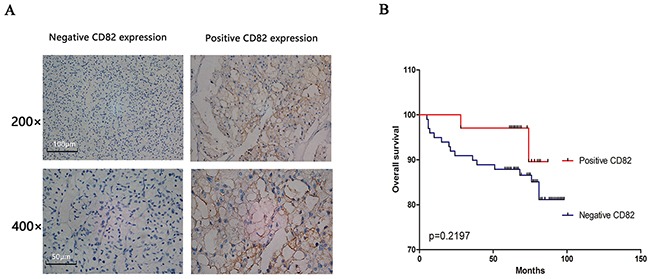
RCC tissues in two human RCC TMAs were used to assess the relationship between CD82 and clinic factors of RCC CD82 was mainly expressed in the cytomembrane of RCC cells and negative/positive CD82 expression of immunohistochemical staining were shown in Figure **(A)**. Positive CD82 protein showed no significance with long overall survival in RCC patients in our study by univariate Kaplan-Meier/log-rank analysis **(B)**.

To further explore the protein expression of CD82 in RCC tissues, 133 RCC tissues from two TMAs were analyzed by immunohistochemical technique. As shown in Figure 5A, CD82 was mainly expressed in the cytomembrane of cancer cells. Characteristics of the 133 RCC patients involved in this study were showed in Table 1 and the positive rate of CD82 was 25.6% (34 of the 133 RCC tissues). We found advanced tumour stage, histological grade and tumor size were significantly associated with CD82 positive expression according to our analysis results. However, no significant differences were revealed between negative and positive expression of CD82 in age, gender and histology (Table 2). Univariate Kaplan-Meier/log-rank analysis was also conducted but showed no significance between positive CD82 protein expression and increased risk for poor clinical prognosis in RCC patients (log rank p=0.2197; Figure 5B).

**Table 1:** Characteristics of the 133 RCC patients involved in this study

Age		
Mean±SD, year	56.34±13.73	
<60	48	36.1%
≥60	85	63.9%
Gender		
Male	83	62.4%
Female	50	37.6%
Tumor size		
Mean±SD, cm	4.81±2.56	
≤4	71	53.4%
>4	62	46.6%
Histology		
Clear cell carcinoma	118	88.7%
Others	15	11.3%
Histological grade		
I	28	21.0%
II	82	61.7%
III	19	14.3%
IV	4	3.0%
Tumor stage		
T1	115	86.5%
T2	11	8.3%
T3	6	4.5%
T4	1	0.8%
Survival		
Mean±SD, month	70.40±19.97	
No	18	13.5%
Yes	115	86.5%
CD82 expression		
Negative	99	74.4%
Positive	34	25.6%

SD, standard deviation

**Table 2:** Relationship of CD82 expression and clinicopathologic characteristics of patients

Variable	Total (%)	CD82 expression		
		Negative (n=99)	Positive (n=34)	*P* value
Age				0.599
<60	48	37 (77.1)	11 (22.9)	
≥60	85	62 (72.9)	23 (27.1)	
Gender				0.929
Male	83	62 (74.7)	21 (25.3)	
Female	50	37 (74)	13 (26)	
Tumor size				0.020
≤4	71	47 (66.2)	24 (33.8)	
>4	62	52 (83.9)	10 (16.1)	
Histology				0.174
Clear cell carcinoma	118	90 (76.3)	28 (23.7)	
Others	15	9 (60)	6 (40)	
Histological grade				0.041
I-II	110	78 (70.9)	32 (29.1)	
III-IV	23	21 (91.3)	2 (8.7)	
Tumor stage				0.036
T1	115	82(71.3)	33(28.7)	
T2-T4	18	17(94.4)	1(5.6)	

Bold: *P* < 0.05. *P* values were two-tailed and based on the Pearson chi-square test.

## DISCUSSION

Given that tetraspanin proteins are likely to function as a part of larger molecular aggregates involved in the control of cell proliferation and migration [14], significant efforts are now being directed towards the identification of specific functional partners for individual tetraspanins. CD82, a member of the tetraspanin protein family, has previously been shown to interfere with cancer proliferation, invasion and migration, and it has been supported by an inverse correlation between its expression levels and the metastases of a variety of human cancers [6, 7]. Yang et al reported that overexpression of CD82 in breast cancer cells resulted in the suppression of *in vitro* invasion and *in vivo* metastasis [15]. Similar effects on migration and invasion abilities of cancer cell lines were found in non-small cell lung cancer, bladder cancer, oral cancer, ect [10, 16, 17]. CD82 can interact with other tetraspanin proteins(eg CD151, CD81), integrins(eg α_3_β_1_, α_4_β_1_, α_5_β_1_) and chemokines to regulate the migration, adhesion and signaling of cells [13, 18, 19]. Integrins are not the only molecules that CD82 regulate and studies have revealed that tetraspanins play a critical role in regulating receptor tyrosine kinase signaling in immune cells [20, 21]. Odintsova et al reported that CD82 was a regulator of epithelial growth factor (EGF)-induced signaling and showed that the association of EGF receptor (EGFR) with the tetraspanin is critical in EGFR desensitization [22].

Although previous studies showed that CD82 functions as a suppressor tumor gene in various carcinomas, the function of CD82 in RCC have not been reported. Our study demonstrated that CD82 could be an important indicator of RCC progression, and it might be involved in RCC metastasis.

We confirmed the down-regulated levels of CD82 in RCC tissues and cell lines by RT-PCR, immunblotting and the immunohistochemistry methods. The clinic-opathological role of CD82 may provide evidence that the loss of CD82 could lead to RCC progression and metastasis. However, our data showed no significance between CD82 expression and increased risk for poor clinical prognosis in RCC patients by univariate Kaplan-Meier/log-rank analysis. The relatively small amounts of patients recruited in our study may have certain influence in this result and further more patients will be included to confirm it. Cell function assay demonstrated that the expression levels of CD82 got an inverse correlation with cell migration/invasion but the proliferation assay in Caki-1 and 786-O cell lines showed no significant difference between the parental and the transfected cells using CCK8 assay (p > 0.05; Supplementary Figure 1). Consequently, we suggested the aberrant expression of CD82 can act as a tumor suppressor and therapeutic target for treating RCC.

Metastasis of cancer cells involves various cytophysiological changes and the degradation of the extracellular matrix(ECM) by increasing the expression and activation of extracellular proteases, such as metalloproteinases (MMPs), urokinase-type plasminogen activator (u-PA), or serine proteinase, has been regarded as important in cancer invasion and metastatic processes [23]. MMPs are the most essential proteases in the proteolysis of ECM proteins(eg collagen, fibronectin, proteoglycan, laminin, and elastin) [24, 25] and they function as important roles in proliferation, differentiation, angiogenesis, and the destruction of inflammatory tissue [26]. Among all the involved proteases, MMP2 and MMP9 are the most essential for degradation of the basement membrane and so they play a major role in cancer invasion and migratio [16, 25]. At this point, we tested the MMPs protein expression of transfected RCC cell lines and our data demonstrated that CD82 could down-regulate the expression of MMP2 and MMP9, thus attenuating the migratory ability of RCC cells. And this is consistent with the previous outcome of cell migration and invasion assays.

There is a growing body of evidence supporting that transforming growth factor-β (TGF-β1) plays a significant role in inducing MMPs through various signaling pathways [23, 27–29]. Therefore, we conjectured if CD82 could affect the expression of TGF-β1 and attenuate the TGF-β1-induced MMPs expression. To verify the assumption, we further tested the protein expression of TGF-β1and its signaling pathway-related biomarkers. Our data showed that overexpression of CD82 notably restrained the protein expression levels of TGF-β1 and the opposite results were found in CD82-knockdown cell line. TGF-β1 acts through its cell surface receptor complexes including TGF-β type I (TβRI) and TGF-β type II (TβRII) receptors, to activate transcription factors Smad 2/3, which regulate TGF-β target gene expression [29–31]. Recruitment and phosphorylation of TβRI phosphorylate intracellular Smad2 and Smad3, which then interact with Smad4 protein to regulate gene expression in the nucleus [27, 31]. When CD82 was interfered or overexpressed artificially in our study, the changes of p-Smad2/3 expressions were consistent with TGF-β1, which revealed that CD82 can exactly play a negative regulation role of TGF-β1/Smad signaling in RCC cell line. However, CD82 did not affect the mRNA level of TGF-β1, as well as downstream Smad2, Smad3, MMP2 and MMP9 (p > 0.05; Supplementary Figure 2), indicating that CD82 might downregulate TGF-β1 through posttranscriptional regulation and a complex mechanism is involved in the CD82/TGF-β1 pathway.

In the present study, the expression of the migration (MMP-2 and MMP-9), TGF-β signaling (TGF-β1, p-Smad2 and p-Smad3) pathways-related proteins were inhibited by CD82 lentiviral treatment in OV group. Further, to confirm whether CD82 attributed its antitumor role to TGF-β1 in RCC or not, we stimulated the OV group cells with Rh TGF-β1 to restore the TGF-β1 expression. TGF-β1 signaling could be revived and CD82-induced inhibitory effect of cell migration/invasion was impaired by the stimulating factor, suggesting that CD82 inhibits the level of TGF-β1activation rather than completely suppressing it.

Our significant finding of this study is the demonstration of a regulatory role of CD82 in TGF-β/Smad pathway which is first detected in RCC cell line. However, further studies are needed to investigate the specific mechanism underlying the regulation and assays *in vivo* are required to further confirm the inhibitory role of CD82 in RCC.

In conclusion, CD82 was down-regulated in RCC and positive CD82 expression was significantly associated with tumour stage, histological grade and tumor size. In addition, we first detected CD82 as a potential tumor suppressor in RCC by inhibiting TGF-β/Smad signaling pathway and play a crucial role in RCC migration and invasion. Therefore, CD82 might serve as a competent candidate for development of new therapeutics against RCC.

## MATERIALS AND METHODS

### Patients and tissue microarrays (TMAs)

Following the Local Ethics Committees of the First Affiliated Hospital of Nanjing Medical University, China, 30 paired tumor specimens and tissue samples used to assess CD82 expression were obtained with informed consent from RCC patients. All the patients had undergone radical nephrectomy or partial nephrectomy. All samples were obtained during surgery, immediately frozen in liquid nitrogen, and stored at –80 °C for further analysis. The identification of tumor tissues and adjacent normal tissues were confirmed by the pathologists.

Two human RCC tissue microarrays (TMAs) containing 133 RCC tissues were obtained from patients who were treated by partial or radical nephrectomy between 2008 and 2011 at the First Affiliated Hospital of Nanjing Medical University (Nanjing, China). All patients were recruited following informed consent and the protocols used in the study was approved by the medical ethics committee of the hospital. The TMA's accompanying pathological data are described in Table 1. The follow-up deadline was April 2016. From each RCC tissue, triplicate tissue cores with diameters of 0.6mm were represented.

### Immunohistochemistry

Serial sections from TMA blocks were deparaffinized in xylene and rehydrated through an ethanol gradient, then were blocked in hydrogen peroxide in methanol for 10 min. Antigen retrieval was performed by incubation for 2 min in a steam pressure cooker containing citrate buffer 10 mM, pH 6.0. Then samples were blocked for 5 min and incubated overnight with primary antibodies against CD82 (Sigma, USA)(1:100) at 4°C overnight. After having been washed by phosphate buffer saline (PBS) for 10 min, slides were cultured in the secondary antibody for 30 min. After a 10 min wash in PBS, the antibody reaction was visualized with a fresh substrate solution containing DAB. The sections were counterstained with hematoxylin, dehydrated, and coverslipped.

### Evaluation of staining

The immunohistochemical staining was evaluated by two experienced pathologists without knowledge of the clinical data separately. The percentage of positive tumour cells was determined in at least five areas at 400×magnification and assigned to one of the following five categories: 0,<5%; 1, 5–25%; 2, 25–50%; 3, 50–75% and 4,>75%. Meanwhile, the intensity of immunostaining was scored as follows: 0, undetected; 1, light yellow; 2, yellow and 3, brown. By multiplying the two scores, the final immunohistochemical scores of RCC tissues for CD82 were: negative expression (<1) and positive expression (1-12).

### Cell culture and cell transfection

The human RCC cell line (caki-1and 786-O), normal human proximal tubular cell (PTC) line (HK-2) were purchased from the Cell Bank Type Culture Collection of the Chinese Academy of Sciences (Shanghai, China), human RCC cell line(caki-2) was purchased from the China Infrastructure of Cell Line Resources. Cells of the Caki-1 and Caki-2 were maintained in McCoy's 5A (Gibco, USA); cells of 786-O were maintained in RPMI 1640 medium; cells of HK-2 were maintained in Dulbecco's modified Eagle's medium (Gibco, USA), all supplemented with 10% fetal bovine serum (FBS, Gibco, USA) within a humidified atmosphere containing 5% CO2 at 37°C.

Two small interfering RNAs (siRNAs) and one scrambled siRNA (Negative Control) were designed and chemically synthesized by GenePharma (Shanghai, China). The sequences of the two siRNAs targeting CD82 were as follow: 5’-GAAGAGGACAACAGCCUUUTT-3’, 5’-AAAGGCUGUUGUCCUCUUCTT-3’, and 5’-CCCAU CCUGACUGAAAGUATT-3’, 5’-UACUUUCAGUCAG GAUGGGTT -3’. The sequences of scrambled siRNA was 5’-UUCUCCGAACGUGUCACGUTT -3’, 5’-ACGUGA CACGUUCGG AGA ATT-3’. 786-O Cells were cultured in 6-well plates at 60-70% confluence on the day before transfection.

According to the manufacturer's instructions, Cell transfection with the siRNA was performed with Lipofectamine 2000 (Invitrogen) and opti-MEM medium. To alleviate toxicity, the medium surrounding the cells was replaced with RPMI 1640 medium 4–6 h after transfection.

The lentiviral vector with overexpression of CD82 and a lentiviral vector alone used as a negative control were constructed by Genechem (Shanghai, China). Cells overexpressing CD82 were defined as OV group, while cells transfected with lentiviral vector alone were defined as NC group. Cells of the Caki-1 was seeded in 6-well plates at 40% confluence on the day before transfection. For viral infection, titrated viral stocks were suitably diluted in complete medium to obtain the desired multiplicity of infection (MOI) and added to Caki-1 cell monolayers. GFP expression could be detected to assess the infection efficiency 3 days after infection. Five days after infection, cells were harvested into two parts. Real-time reverse transcription polymerase chain reaction (RT-PCR) was performed to evaluate CD82 expression efficiency in one part of cells, while the other part was used for future cell amplification and experiments.

### RNA extraction, RT- PCR and quantitative RT-PCR

The RNA was extracted from cell lines and tumour samples using Trizol reagent (Invitrogen, USA) and spectroscopy was used to detect the purity and concentration of the RNA samples. Total RNA was reversed transcribed into cDNA using PrimeScript RT Master Mix (Takara, JPN). Analysis of CD82 expression was performed by qRT-PCR using a SYBR Green assay in accordance with the manufacturer's instructions (Applied Biosystems, USA). For PCR we used the following primers (Realgene, Nanjing, China): CD82 forward, 5’-GCTCATTCGAGACTACA ACAGC-3’ and reverse, 5’-GTGACCTCAGGGCGAT TCA-3’; β-actin forward, 5’-TGACGGGGTCACCCA CACTGTGCCCATCTA-3’ and reverse, 5’-CTAGAA GCATTTGCGGTGGACGATGGA GGG-3’. Relative expression of CD82 were calculated using the 2^−ΔΔCt^method. Each reaction was run in triplicate.

### Protein isolation and Western blot

Cells and human RCC tissues were lysed in radioimmunoprecipitation assay (RIPA) buffer (Beyotime, Beijing, China), supplemented with protease inhibitors (Roche, Shanghai, China) and the serine protease inhibitor phenylmethylsulfonyl fluoride (PMSF; Roche), at 4°C for 30min. The cell supernatants were extracted after centrifugation for 15 min at 14,000 rpm, then the concentration of the protein was determined using a BCA Protein Quantifcation kit (Beyotime Institute of Biotechnology). Proteins from tissues and cells were separated using 10% SDS-PAGE, transferred onto PVDF membranes (Millipore, Billerica, USA), blocked for 2h with 5% nonfat milk at room temperature, and incubated with primary antibodies at 4 °C overnight. After that, the membranes were washed three times with TBST and incubated with a horseradish peroxidase-conjugated secondary antibody for 2 h at room temperature. Blots were detected using a Bio-Rad Bioimaging system (Bio-Rad, CA, USA), and antibodies against β-tubulin served as a negative control. Rabbit monoclonal antibodies (1:1000) against TGF-β, Smad2/3, p-Smad2, p-Smad, matrix metallopeptidase 2 (MMP2) and matrix metallopeptidase 9 (MMP9) (Cell Signaling Technology, USA) were used in Western blot analysis according to the manufacturer's instructions.

### Transwell migration and invasion assays

For the migration assays, 2×10^4^ cells in 200μl of serum-free medium were placed in the top chamber of the transwell (pore size, 8 mm; BD Bioscience). For the invasion assays, 5×10^4^ cells in 200μl of serum-free medium were placed in the top chamber adhered with Matrigel (BD Bioscience) following the manufacturer's protocol. While the lower chamber was filled with 20% FBS-containing medium. After incubation at 37°C for 24 hours, the cells on the upper surface were erased by a cotton swab, while the cells invaded on the bottom of membranes were fixed in 4% methanol solution and stained with 0.5% crystal violet and counted under a microscope. Five fields were randomly counted. Experiments were repeated 3 times.

### Recombinant human TGF-β 1 stimulation

Recombinant Human TGF-β 1(Rh TGF-β 1) (R&D system, USA) was reconstituted at 20 µg/mL in sterile 4 mM HCl containing 0.1% bovine serum albumin for storing and use. The stimulating concentration to the OV group cells was 2ng/ml when cells were seeded in 6-well plates at 50% confluence. Cells were harvested for further transwell assays and protein isolation 48h after the stimulation. OV group cells stimulated with Rh TGF-β 1 were defined as OV+TGF-β 1 group.

### Statistical analyses

We used χ^2^-test to evaluated the associations between CD82 protein expression and clinicpathological factors. The association between CD82 expression and overall survival was assessed by Kaplan–Meier curve and log-rank tests. All data values are expressed as mean ± SD and statistical analyses were performed using the SPSS20.0 software. P < 0.05 was considered statistically significant.

## SUPPLEMENTARY MATERIALS FIGURES


