# Supplementary material for: The metastasis suppressor CD82/KAI1 regulates cell migration and invasion via inhibiting TGF-β 1/Smad signaling in renal cell carcinoma
Source: Oncotarget. 2017 May 23;8(31):51559–68. doi: 10.18632/oncotarget.18086 (PMC5584269; doi:10.18632/oncotarget.18086)
Supplement: Supplementary file 1 [file oncotarget-08-51559-s001.pdf]

The metastasis suppressor CD82/KAI1 regulates cell migration and invasion via inhibiting TGF-β 1/Smad signaling in renal cell carcinoma

SUPPLEMENTARY MATERIALS

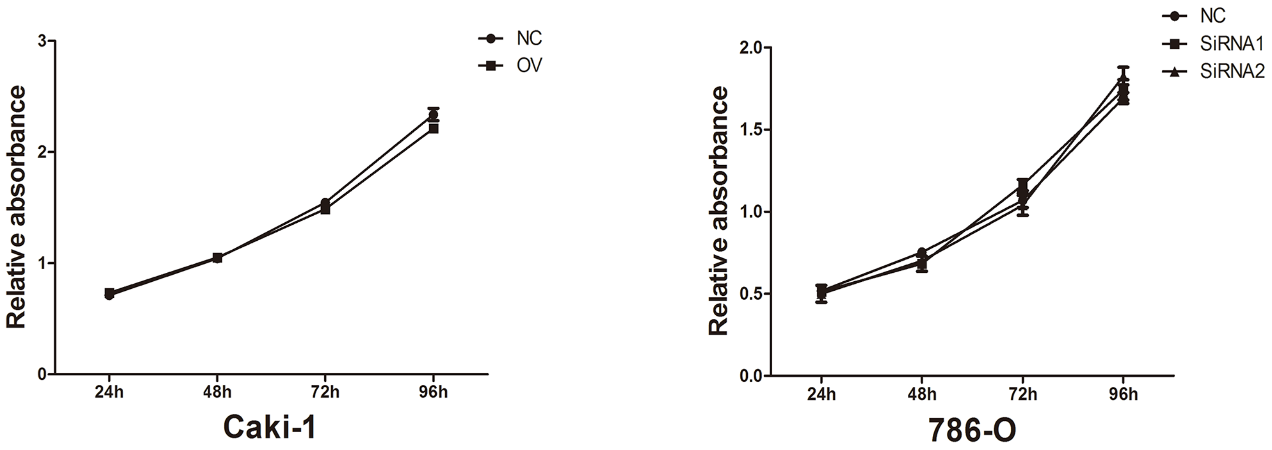

Supplementary Figure 1: CCK8 assays conducted in transfected Caki-1 and 786-O cells.

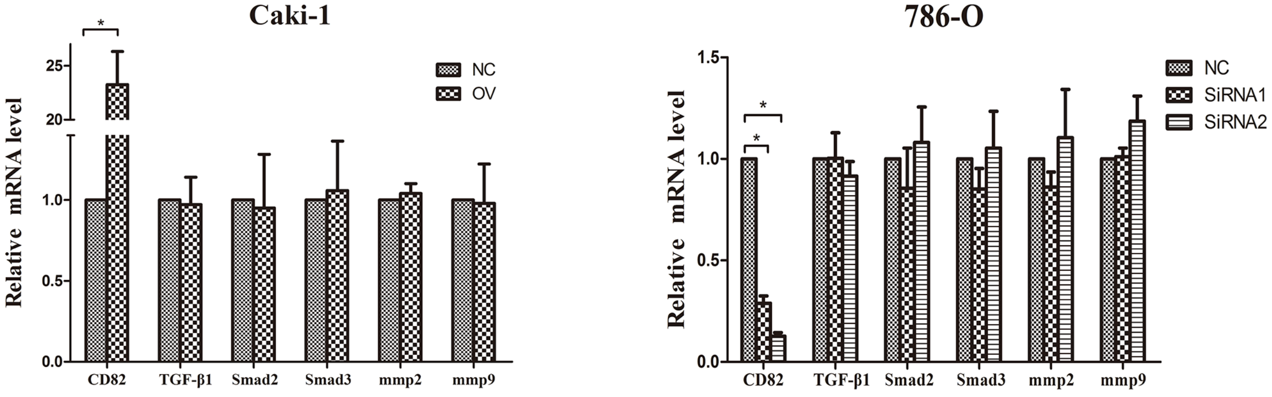

Supplementary Figure 2: The mRNA expression of TGF-β1, Smad2, Smad3, MMP2 and MMP9 in transfected Caki-1 and 786-O cells.
